# Two New Stilbenoids from the Aerial Parts of *Arundina graminifolia* (Orchidaceae)

**DOI:** 10.3390/molecules21111430

**Published:** 2016-10-27

**Authors:** Florence Auberon, Opeyemi Joshua Olatunji, Stéphanie Krisa, Cyril Antheaume, Gaëtan Herbette, Frédéric Bonté, Jean-Michel Mérillon, Annelise Lobstein

**Affiliations:** 1 Laboratory of Pharmacognosy and Bioactive Natural Products, Faculty of Pharmacy, Strasbourg University, Illkirch-Graffenstaden 67400, France; pere@fastermail.com (O.J.O.); lobstein@unistra.fr (A.L.); 2 GESVAB Group, Oenology Research Unit, EA 4577, USC 1366 INRA, ISVV, Faculty of Pharmacy, Bordeaux University, Villenave d’Ornon 33140, France; stephanie.krisa@u-bordeaux.fr (S.K.); jean-michel.merillon@u-bordeaux.fr (J.-M.M.); 3 Laboratoire Insulaire du Vivant et de l’Environnement, EA 4243, New-Caledonia University, BP R4, Noumea CEDEX 98851, New Caledonia; cyril.antheaume@univ-nc.nc; 4 Spectropôle, FR1739, Aix-Marseille University, Campus de St Jerome-Service 511, Marseille 13397, France; gaetan.herbette@univ-amu.fr; 5 Louis Vuitton Moët et Hennessy Recherche, 185 avenue de Verdun, St Jean de Braye 45800, France; fredericbonte@research.lvmh-pc.com

**Keywords:** *Arundina graminifolia*, Orchidaceae, phenanthrene derivatives, arundiquinone, arundigramin

## Abstract

Two new phenanthrene derivatives, a phenanthrenequinone named arundiquinone (**1**) and a 9,10-dihydrophenanthrene named arundigramin (**2**) together with a known lignin dimer (**3**) and seven known stilbenoids (**4**–**10**) were isolated from the aerial parts of the Asian orchid *Arundina graminifolia*. The structures of the isolated compounds were elucidated by spectroscopic methods, including extensive 1D, 2D NMR (heteronuclear single quantum coherence (HSQC), heteronuclear multiple-bond correlation spectroscopy (HMBC), and HR-ESI-MS techniques, as well as comparison with respective literature reports. The cytoprotective activity of the isolated compounds were evaluated for their ability to reduce beta amyloid induced toxicity on undifferentiated PC12 cells. Compound **8** showed moderate cytoprotective activity at 0.5 µmol/L (71% of cell viability) while the other compounds showed no significant activity at the highest concentration tested.

## 1. Introduction

*Arundina graminifolia* (D. Don) Hochr. (Orchidaceae), known as the bamboo orchid, is an evergreen terrestrial growing orchid and the sole accepted species in the genus *Arundina*. It is widely distributed in Southeast Asia, from the Himalayas to western Indonesia [1]. The whole plant is mainly used in Chinese traditional Dai medicine as a treatment for blood stasis, food poisoning and as a liver detoxifying agent [2,3,4,5]. It is also used as an antibacterial and emollient in India [6,7] and for the treatment of rheumatism in Bangladesh [8]. Its striking purple flowers are the reason for its harvest for ornamental purposes [9]. Stilbenoids are the major secondary metabolites reported in this orchid based on previous phytochemical studies, revealing an important structural diversity of diphenylethylenes [10,11,12,13,14], bibenzyls [15,16,17,18], phenanthrenes [19], 9,10-dihydrophenanthrene derivatives [20] and other phenolic compounds [21,22,23,24,25,26], and thus could be considered as potential chemotaxonomic markers for the genus. These metabolites are also known to display a wide range of biological activities such as antioxidant, antiviral, cytotoxic and antitumoral properties [27,28,29,30,31,32].

As part of our continuing efforts in contributing to the phytochemical and biological evaluation of tropical orchids [33,34,35], the ethyl acetate extract of the aerial parts of *A. graminifolia* collected from Chiang Mai Province (Thailand) was screened for its neuroprotective activity against beta amyloid (βA) induced cytotoxicity on PC12 cells and showed promising results. Based on the aforementioned preliminary screening, the extensive investigation on the chemical entities in the plant was pursued. Two new constituents, 1–4 phenanthrenequinone (**1**) and 9,10-dihydrophenanthrene (**2**) along with a known lignan dimer (**3**) and seven known stilbenoids (**4**–**10**) were isolated and characterized. The cytoprotective activity of the compounds was then assessed to establish whether or not it is responsible for the cytoprotective activity of the tested extract.

## 2. Results and Discussion

**Figure 1:**
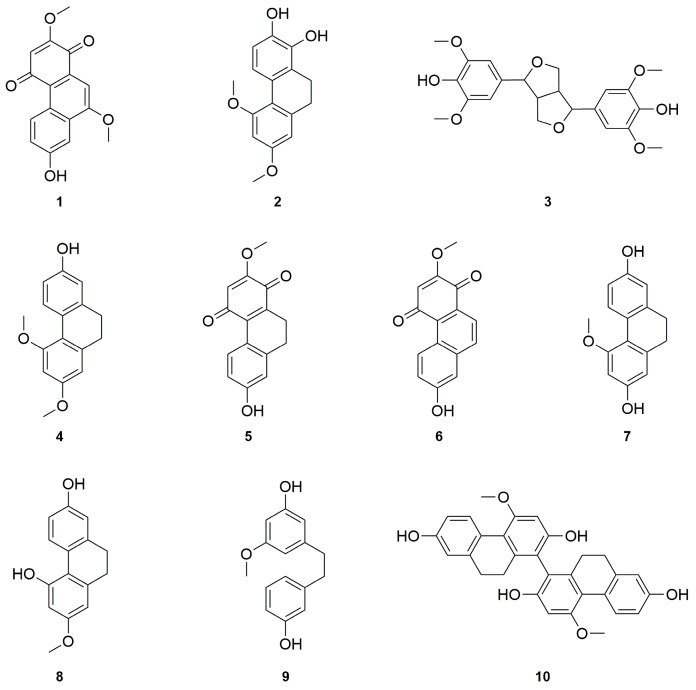
Chemical structures of compounds **1**–**10** isolated from *A. graminifolia* aerial parts.

The ethyl acetate (EtOAc) extract of the aerial parts of *A. graminifolia* was subjected to a series of chromatographic techniques (silica gel, Sephadex LH-20, Sigma Aldrich, Saint-Louis, MO, USA), and semi-preparative RP-HPLC) to afford two new stilbenoids: arundiquinone and arundigramin (**1** and **2**), as well as eight known compounds that were identified by comparison of their spectroscopic data to previously published reports as *rac*-syringaresinol (**3**) [36,37], orchinol (**4**) [38], ephemeranthoquinone (**5**) [39], densiflorol B (**6**) [40], coelonin (**7**) [41], lusianthridin (**8**) [42], batatasin III (**9**) [43], and flavanthrin (**10**) [44], (Figure 1). It is noteworthy to add that, besides the two new isolated stilbenoids (**1** and **2**), compound **3** syringaresinol is herein reported as its first occurrence in *A. graminifolia*.

Compound **1** was obtained as a red amorphous powder. Its molecular formula was determined to be C_16_H_12_O_5_ based on the molecular ion peak at *m*/*z* 285.0760 [M + H]^+^ (calcd for C_16_H_13_O_5_, 285.0758) as observed in the HR-ESI-MS, which corresponds to eleven degrees of unsaturation. The UV spectrum of **1** showed absorption maxima at 213, 247, 301, 310, 390 and 498 nm, which were similar to those of the phenanthrenequinones [45,46]. The phenolic and quinone moiety in compound **1** was also supported by the IR absorptions at 3292 cm^−1^ (hydroxyl), 1670 cm^−1^ (carbonyl) and 1605, 1585, 843, and 797 cm^−1^ (aromatic ring). The ^13^C-NMR and HSQC spectra revealed the presence of 16 carbon resonances including nine quaternary carbons, four methines and two methoxy groups. Among the nine quaternary carbons, two were carbonyl carbons according to their chemical shifts at δ_C_ 181.4 (C-1) and 188.5 (C-4). The ^1^H-NMR spectrum of **1** (Table 1) exhibited the presence of an ABX spin system as observed in the aromatic protons at δ_H_ 9.50 (1H, d, *J* = 9.4 Hz, H-5), 7.36 (1H, dd, *J* = 9.4, 2.3 Hz, H-6) and 7.63 (1H, d, *J* = 2.3 Hz, H-8), which indicated the presence of a tri-substituted aromatic ring. Additional signals belonging to two isolated aromatic protons at δ_H_ 6.10 (1H, s, H-3) and 7.42 (1H, s, H-10), and two methoxy at δ_H_ 3.91 (3H, s, 2-OCH_3_) and 4.15 (3H, s, 9-OCH_3_) were also noticed. The HMBC cross peaks (T) were observed from H-3 to C-1, C-2, C-4 and C-4a; H-5 to C-4a, C-6, C-7 and C-8a; H-6 to C-4b and C-8; H-8 to C-4b, C-6 and C-9 and H-10 to C-1, C-4a, C-8a and C-9. On the basis of the above evidence, the skeleton of **1** was confirmed as a phenanthrene-1,4-dione. The positions of the methoxy and hydroxyl groups were established by HMBC and NOESY (Nuclear Overhauser Effect Spectroscopy) correlations. HMBC cross peaks were observed between 2-OCH_3_ to C-2 and 9-OCH_3_ to C-9 indicating the position of the two methoxy groups on C-2 and C-9. This was further supported by NOESY correlations from 2-OCH_3_ to H-3, and 9-OCH_3_ to H-8 and H-10. The remaining substituent, which is the hydroxyl group, was thus substituting on C-7. This was supported by the characteristic chemical shift on an oxygenated carbon (δ_C_ 158.7). The structure of compound **1** was established as 7-hydroxy-2,9-dimethoxy-1,4-phenanthrenequinone named arundiquinone. The ^1^H-NMR, ^13^C-NMR, HSQC, HMBC, NOESY and HRESIMS spectrum of compounds (**1**) and (**2**) are available at Figures S1–S12.

**Figure 2:**
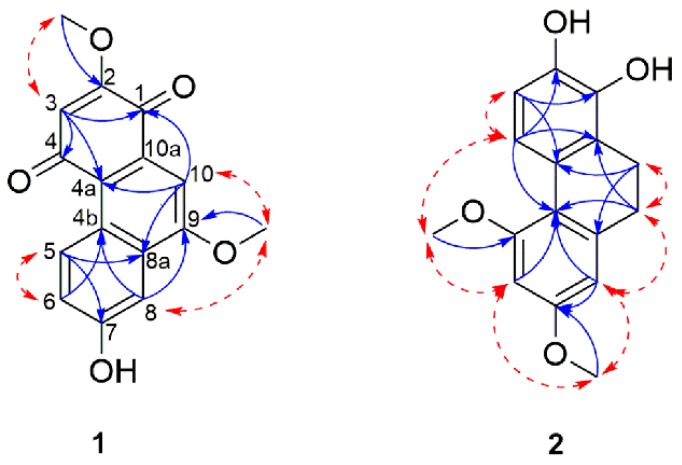
NOESY (**red** dashed arrows) and HMBC (**blue** arrows) correlations of compounds **1** and **2**.

Compound **2** was obtained as a pale rose amorphous solid. The HR-ESI-MS [M + H]^+^ at *m*/*z* 273.1138 supported the molecular formula of C_16_H_16_O_4_ (calcd for C_16_H_17_O_4_, 273.1090), which indicated nine degrees of unsaturation. The UV spectrum exhibited three maxima at 219, 282 and 307 nm, which were similar to 9,10-dihydrophenanthrene derivatives [47,48]. The IR spectrum showed broad absorption bands at 3366 cm^−1^ for hydroxyl groups and 1588, 163, 1063, 797 cm^−1^ for aromatic rings. Analysis of the ^13^C-NMR and HSQC spectra revealed the presence of eight quaternary carbons, four aromatic methines carbons, two methoxy carbons and two methylene carbons. The ^1^H-NMR data (Table 1) showed signals belonging to a pair of *ortho*-coupled protons at δ_H_ 6.70 (1H, d, *J* = 7.3 Hz, H-3) and 7.65 (1H, d, *J* = 7.3 Hz, H-4), two *meta*-coupled aromatic protons at δ_H_ 6.51 (1H, d, *J* = 2.4 Hz, H-6) and 6.47 (1H, d, *J* = 2.4 Hz, H-8), two methylenes at δ_H_ 2.67 (2H, m, H-9) and 2.74 (2H, m, H-10) and two methoxy groups δ_H_ 3.85 (3H, s, 5-OCH_3_) and 3.81 (3H, s, 7-OCH_3_). The HMBC correlations (Figure 2) of the methoxy at δ_H_ 3.85 to C-5 and δ_H_ 3.81 to C-7 confirmed that the methoxy groups were located on C-5 and C-7, respectively. This was also supported by NOESY correlations from 5-OCH_3_ to H-4 and H-6 as well as from 7-OCH_3_ with H-6 and H-8. The remaining two hydroxyl groups were thus assigned to C-1 and C-2 with the help of the HMBC cross peaks correlations between H-3 and H-4, respectively, to the last two oxygenated quaternary carbons C-1 and C-2. Thus, the structure of compound **2** was defined as 5,7-dimethoxy-9,10-dihydrophenanthrene-1,2-diol, named arundigramin.

**Figure 3:**
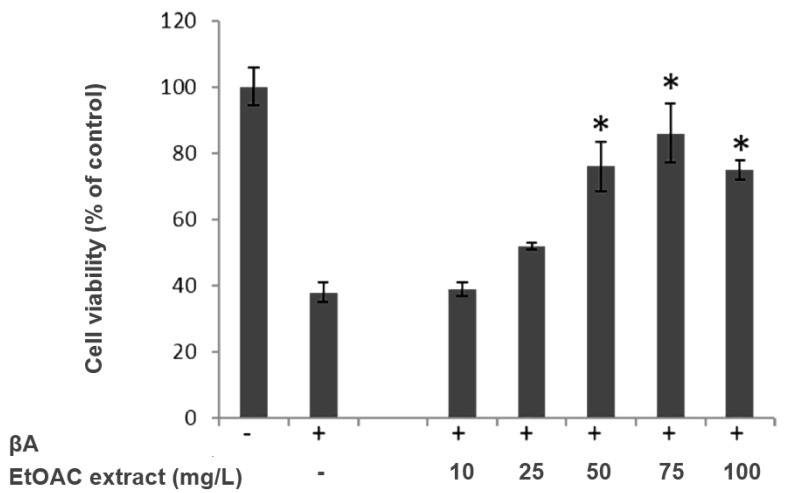
Effect of the EtOAc extract on PC12 cell viability. PC12 cells were incubated with EtOAc extract (10, 25, 50 and 100 mg/L) for 24 h, and then exposure to 5 μM of βA for 24 h. Results are expressed as mean ± SD (*n* = 4). * *p* < 0.05 as compared to the βA treated group.

**Figure 4:**
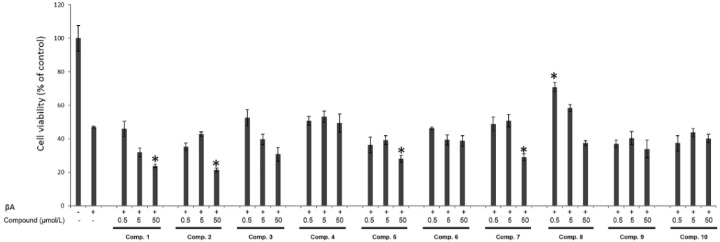
Effect of compounds **1**–**10** on PC12 cell viability. PC12 cells were incubated with compounds **1**–**10** (0, 5 and 50 μM) for 24 h, and then exposure to 5 μM of βA for 24 h. Data are expressed as mean ± SD (*n* = 4). * *p* < 0.05 as compared to the βA treated group.

Cell viability, expressed as a percentage relative to the untreated control cells, decreased by more than 60% after exposure to βA_25–35_ alone. The EtOAc extract of *A. graminifolia* aerial parts showed promising cytoprotective activity against βA induced cytotoxicity on undifferentiated PC12 cells (86% cell viability at 75 mg/L) (Figure 3). Despite this effect, the isolated compounds (**1**–**10**) were tested for their potential cytoprotective effect. Only compound **8** displayed moderate cytoprotective activity at 0.5 µmol/L, and the other compounds did not exhibit any significant effect at the tested concentrations (Figure 4). Furthermore, compounds **1**, **2**, **3**, **5** and **7** were cytotoxic to the cells at the highest tested concentration (50 µmol/L).

## 3. Experimental Section

### 3.1. General Experimental Procedures

Optical rotations were measured with a Perkin Elmer 341 polarimeter (Perkin-Elmer Inc., Waltham, MA, USA). UV spectra were recorded on a Shimadzu UV-2401 PC spectrometer (Shimadzu, Kyoto, Japan). IR spectra were obtained on a 380 FT-IR spectrophotometer (Thermo Fisher Scientific, Waltham, MA, USA). The 1D and 2D NMR spectra were performed on a 500 MHz proton operating system on a Bruker Avance III spectrometer (Bruker BioSpin, Rheinstetten, Germany) Acetone-*d*_6_ (Euriso-Top, Saint-Aubin, France) was used as deuterated solvent and its protonated residual signal was used as internal standard at 2.05 ppm relative to TMS. The HR-ESI-MS analyses were performed on an HPLC-DAD/UV-MS Agilent 1200 Series coupled to a 6520 Q-ToF mass spectrometer (Agilent Technologies, Santa Clara, CA, USA). The acquisition of mass spectra was conducted in ESI positive ion mode. A Varian LC-920 HPLC-DAD/UV system (Varian Inc., Palo-Alto, CA, USA) equipped with a Kinetex XB-C18 column (100 mm× 3.0 mm i.d, 2.6 µm) (Phenomenex, Torrance, CA, USA) was used for HPLC-DAD/UV analysis. The prepacked Solid Phase Extraction (SPE) Chromabond cartridge (SiOH, 50 g/150 mL) was purchased from Macherey–Nagel (Macherey–Nagel, Düren, Germany), and SPE fractions were monitored by TLC. The spots were visualized either under UV light (254 nm) and under visible light after heating the plates sprayed with 2% sulfuric vanillin reagent. Sephadex LH-20 (Sigma Aldrich) was used for gel chromatography eluting with methanol. Semi-preparative RP-HPLC experiments were conducted on a Gilson LC system (Gilson Inc., Limburg an der Lahn, Germany) equipped with a semi preparative Kinetex Axia C-18 Column (100 mm × 21.2 mm i.d, 5 µm) (Phenomenex, Torrance, CA, USA). Analytical TLC plates were carried out on pre-coated alumina silica gel 60F_254_ plates (0.25 mm thickness) (Merck, Darmstadt, Germany). Analytical grade solvents of HPLC quality were purchased from Sigma Aldrich.

### 3.2. Plant Material

The dried aerial parts (stems and leaves) of *A. graminifolia* (D. Don) Hochr. (Orchidaceae) of flowering specimens were purchased in September 2010 at Joe’s Orchid Farm in Chiang Mai Province, Thailand and imported to France in compliance with the Convention on International Trade of Endangered Species (CITES). A voucher specimen (No. 05-563) was deposited at the herbarium of the Faculty of Science Chiang Mai University, Chiang Mai, Thailand.

### 3.3. Extraction and Isolation

The air-dried powder of the aerial parts of *A. graminifolia* (100 g) was subjected to successive extraction using cyclohexane, EtOAc and CH_3_OH. Each extraction was performed by maceration for 30 min followed by sonicating in an ultrasonic bath for 10 min at room temperature (1 g raw material per 15 mL of organic solvent) and filtered. Extractions were repeated three times, and the filtrates were combined and evaporated under reduced pressure to afford cyclohexane extract (0.61 g), EtOAc extract (2.49 g) and CH_3_OH extract (11.01 g).

The EtOAc extract (2.49 g) was subjected to a normal phase SPE cartridge (50 g/150 mL) eluting with CHCl_3_/EtOAc (100:0 to 0:100) and EtOAc/CH_3_OH (100:0 to 0:100) to afford 26 fractions (A to Z). Fraction C (67 mg) was subjected to Sephadex LH-20 using CH_3_OH as eluent to give compound **3** (20 mg). Fraction D (29.5 mg) was purified using a semi-preparative RP-HPLC-DAD system (55% (B) for five min, 55%–70% (B) for 11 min, 70% (B) for two min, 70%–100% (B) for two min, 100% (B) for five min, with solvent B (CH_3_OH + 0.05% Formic Acid) and solvent A (water + 0.05% Formic Acid), flow rate 28 mL/min, UV monitoring at λ = 280 nm) to obtain compounds **1** (0.8 mg), **2** (1.9 mg), **3** (1.3 mg), **4** (1.3 mg), **5** (1.2 mg) and **6** (1.3 mg). Fraction F (70 mg) was also purified using the same semi-preparative HPLC method stated above to obtain compounds **7** (4.9 mg), **8** (1.7 mg) and **9** (0.8 mg). Fraction G (41 mg) was subjected to Sephadex LH-20 (CH_3_OH) to afford compound **10** (1.3 mg).

### 3.4. Compound Characterization

Arundigramin (**1**). Red amorphous powder (1.2 mg); UV (CH_3_OH) λ_max_ (log ε): 213 (3.77), 247 (3.53), 301 (3.42), 310 (3.45), 390 (2.56); 498 (2.55); IR (FT-IR) ν_max_: 3292, 2925, 2852, 1670, 1605, 1465, 1353, 1285, 1243, 1110, 1078, 1019, 843, 797 cm^−1^; ^1^H-NMR and ^13^C-NMR see Table 1; HR-ESI-MS: *m*/*z* 285.0759 [M + H]^+^ (calcd. C_16_H_13_O_5_ for 285.0757).

Arundigramin (**2**). Rose pale amorphous powder (1.9 mg); UV (CH_3_OH) λ_max_ (log ε): 219 (4.02), 282 (4.31), 307 (3.93); IR (FT-IR) ν_max_: 3366, 2923, 1588, 1453, 1258, 1156, 1063 and 797 cm^−1^; ^1^H-NMR and ^13^C-NMR: see Table 1; HR-ESI-MS *m*/*z* 273.1138 [M + H]^+^ (calcd. for C_16_H_17_O_4_ 273.1049).

### 3.5. Cytoprotective Assay

#### 3.5.1. Chemical and Reagents

Dimethyl sulfoxide (DMSO), Dulbecco’s modified eagle’s medium (DMEM-Glutamax), thiazolyl blue tetrazolium bromide (MTT), fetal horse serum and fetal bovine serum were purchased from Sigma-Aldrich (Steinheim, Germany). Rat pheochromocytoma cells (PC12 cells) were supplied from the American Type Culture Collection (ATCC^®^, Manassas, VA, USA) and Amyloid beta-protein 25–35 (βA_25–35_) by Synvec (Bordeaux, France).

#### 3.5.2. Cytoprotective Protocol

Undifferentiated PC12 cells were grown in DMEM-Glutamax supplemented with 100 IU/mL of penicillin, 100 µg/mL of streptomycin, 15% fetal horse serum, and 2.5% fetal bovine serum at 37 °C in a humidified atmosphere of 5% CO_2_. Cells were seeded at a density of 2 × 10^4^ cells/well in 96-well culture plates. After 24 h, cells were incubated with the EtOAc extract (10, 25, 50, 75 and 100 mg/L) and compounds **1**–**10** were screened at a concentration of 0.5, 5 and 50 µmol/L in the presence or absence of βA_25–35_ (5 µM). The extract and compounds were dissolved in DMSO at a final concentration of 0.1%.

The cell viability was determined by the colorimetric MTT reduction assay. After treatment (24 h), PC12 cells were incubated with 0.5 mg/mL (DMEM) of MTT for 3 h at 37 °C. The resulting dark blue formazan crystals were dissolved with 100 µL of DMSO. Absorbance values were read at 540 nm on a microplate reader (MRX Dynex, Dynex Technologies, Denkendorf, Germany) [49]. Cell viability was expressed as a percentage of control cells at 100% viability.

Statistical analysis was performed using GraphPad Prism (version 7.0, GraphPad Software Inc. San Diego, CA, USA). All data are expressed as mean ± SD. Data were analyzed using one-way analysis of variance (ANOVA) followed by post hoc analysis using Dunnett’s multiple test. Differences were considered significant at *p* < 0.05.

## 4. Conclusions

Two new phenanthrene derivatives, arundiquinone (**1**) and arundigramin (**2**) together with eight known compounds (**4**–**10**) were isolated from the EtOAc extract of *A. graminifolia* aerial parts, a well-studied Asian orchid. The structures of the new phenanthrenes were elucidated by means of NMR and HR-ESI-MS, as well as comparisons with previous literature reports. Compound **3** was isolated here from *A. graminifolia* for the first time, whereas the presence of compounds **4**–**10** was already signaled in this species. Cytoprotective activity of the isolated compounds was evaluated on their ability to reduce beta amyloid induced toxicity on undifferentiated PC12 cells; however, only compound **8** showed moderate activity while the other isolated compounds did not display any significant activity at the tested concentrations. The EtOAc extract is particularly rich and contains more than 40 compounds based on the HPLC-DAD/UV profiling of this extract and only ten compounds have been isolated and tested. We thus envisage that some minor components that we have yet to isolate may have contributed to the potent bioactivity of the EtOAc extract. Further experiments geared towards identifying these minor components that may be responsible for the displayed cytoprotective activity in the EtOAc extract is in progress.

## Figures and Tables

**Table 1:** NMR spectral data of compounds **1** and **2** (in acetone-*d*_6_, 500 MHz for ^1^H, 125 MHz for ^13^C).

No.	Compound 1			Compound 2		
	δ_H_ (*J* in Hz)	δ_C_	HMBC	δ_H_ (*J* in Hz)	δ_C_	HMBC
1		181.4			141.9	
2		159.3			143.9	
3	6.10 (s)	111.6	1, 2, 4, 4a	6.70 (d, 7.3)	112.8	1, 4a
4		188.5		7.65 (d, 7.3)	120.8	2, 3, 4a, 10a
4a		121.8			126.3	
4b		126.6			117.8	
5	9.50 (d, 9.4)	131.2	4a, 6, 7, 8a		158.7	
6	7.36 (dd, 9.4, 2.3)	122.7	4b, 8	6.51 (d, 2.4)	98.4	4b, 5, 7, 8
7		158.4			159.5	
8	7.63 (d, 2.3)	105.1	4b, 6, 9	6.47 (d, 2.4)	106.0	4b, 6, 7, 9
8a		131.5			141.3	
9		158.9		2.67 (m)	31.3	4b, 8, 8a, 10, 10a
10	7.42 (s)	100.4	1, 4a, 8a, 9	2.74 (m)	22.4	8a, 9, 10a
10a		131.5			125.6	
2-OCH_3_	3.91 (s)	56.7	2			
5-OCH_3_				3.85 (s)	55.9	5
7-OCH_3_				3.81 (s)	55.6	7
9-OCH_3_	4.15 (s)	56.6	9			

## Supplementary Materials

Supplementary materials can be accessed at: http://www.mdpi.com/1420-3049/21/11/1430/s1.
