# Supplementary material for: Two New Stilbenoids from the Aerial Parts of Arundina graminifolia (Orchidaceae)
Source: Molecules. 2016 Oct 27;21(11):1430. doi: 10.3390/molecules21111430 (PMC6273616; doi:10.3390/molecules21111430)
Supplement: Supplementary file 1 [file molecules-21-01430-s001.pdf]

Supplementary Materials: Two New Stilbenoids from the Aerial Parts of *Arundina graminifolia* (Orchidaceae)

Florence Auberon, Opeyemi Joshua Olatunji, Stéphanie Krisa, Cyril Antheaume, Gaëtan Herbette, Frédéric Bonté, Jean-Michel Mérillon and Annelise Lobstein

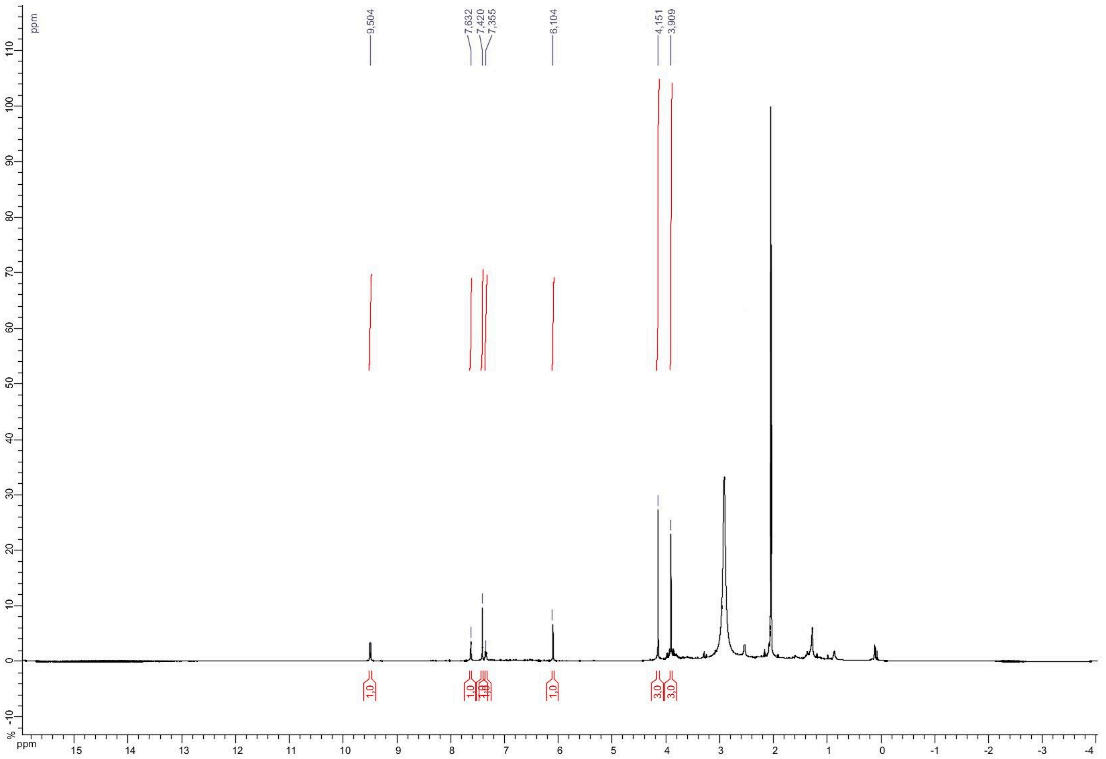

Figure S1. ^{1}H-NMR (500 MHz, acetone-d_6) spectrum of arundiquinone (**1**).

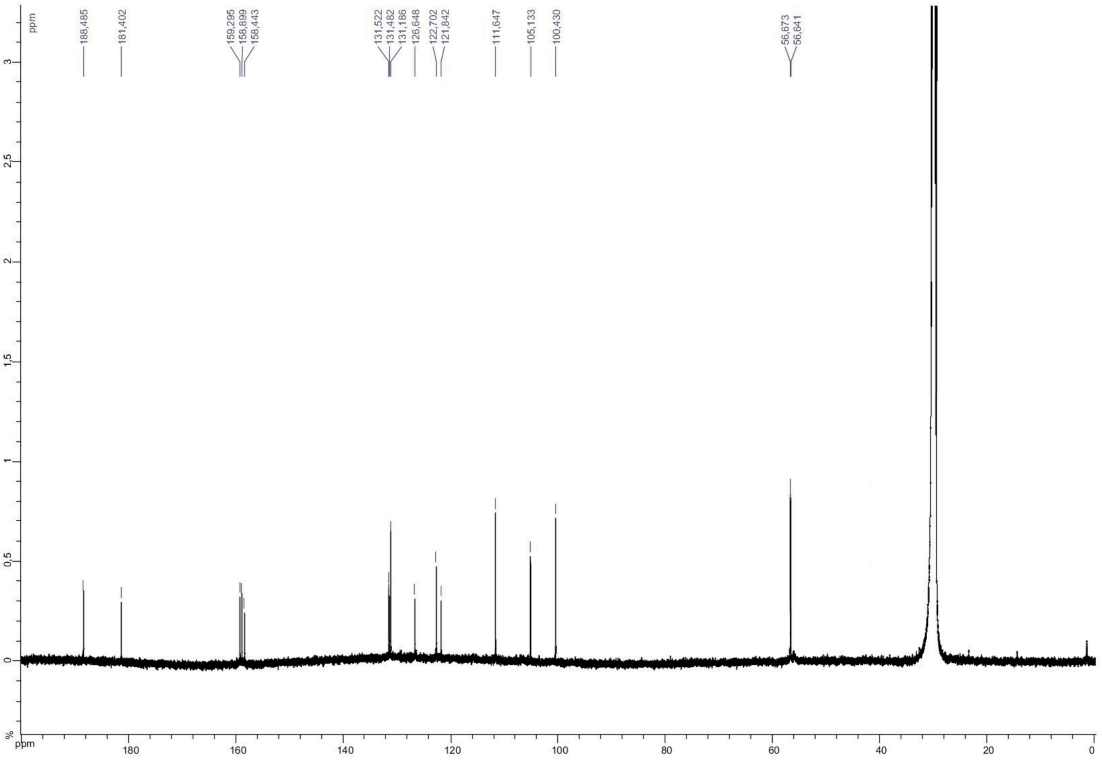

Figure S2. ^{13}C-NMR (125 MHz, acetone-d_6) spectrum of arundiquinone (**1**).

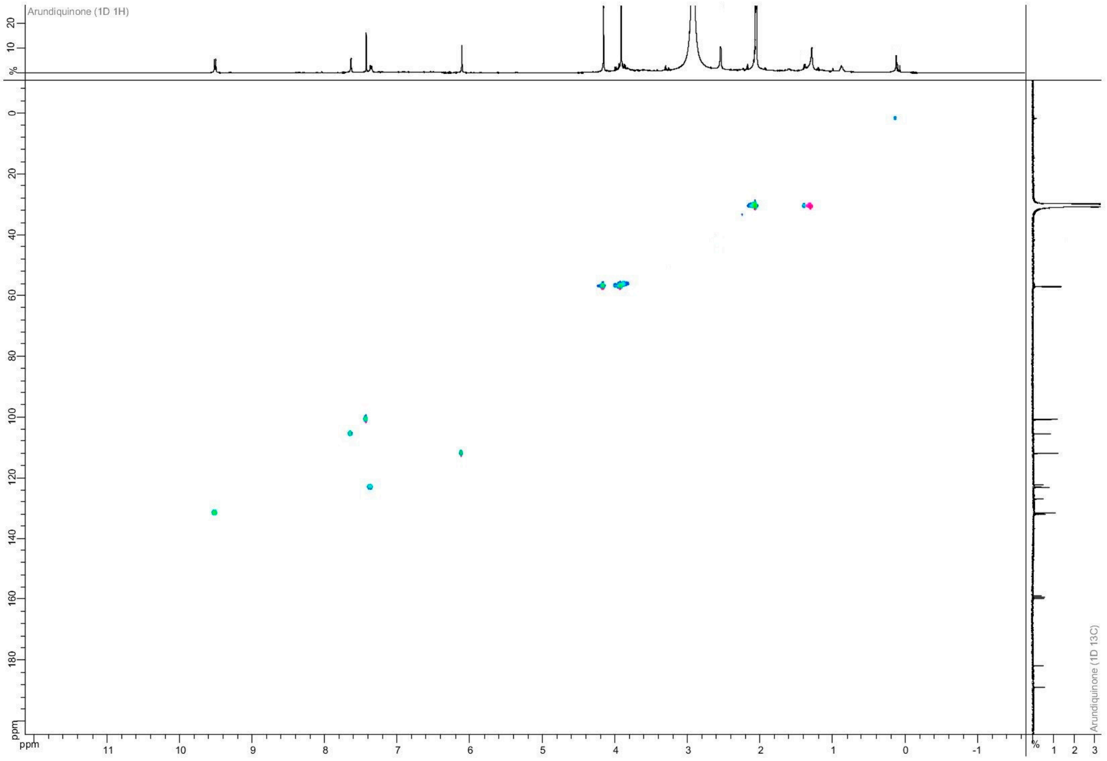

Figure S3. HSQC spectrum of arundiquinone (**1**).

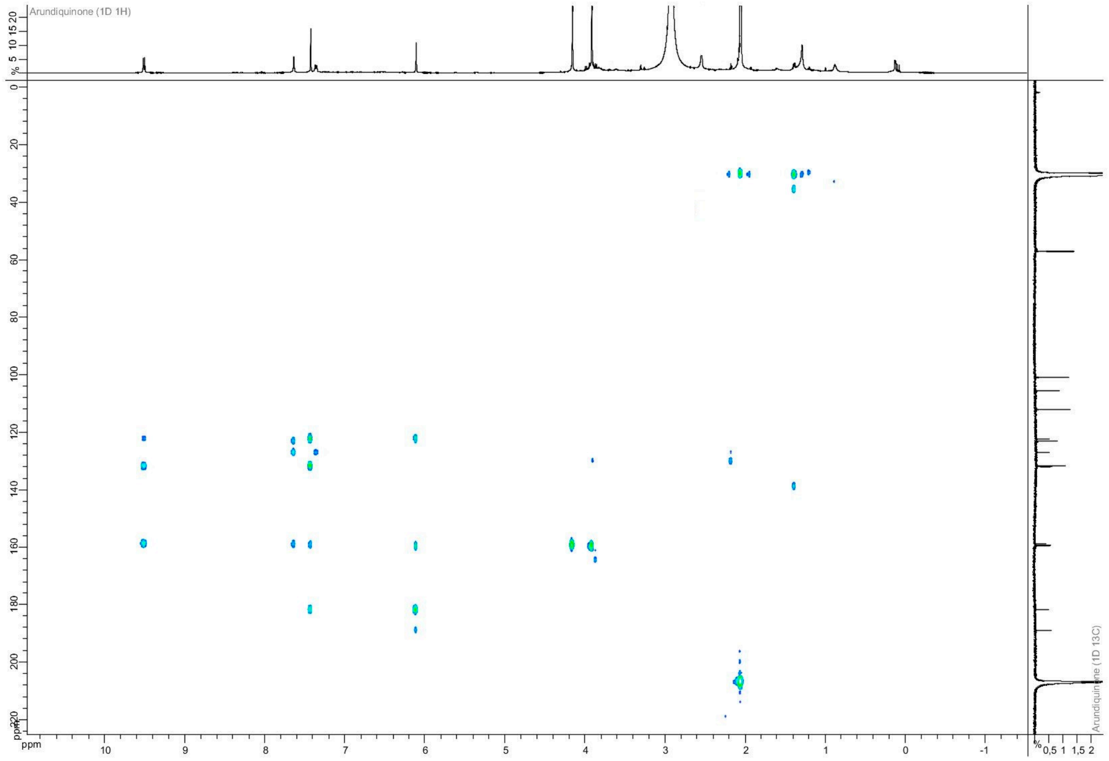

Figure S4. HMBC spectrum of arundiquinone (**1**).

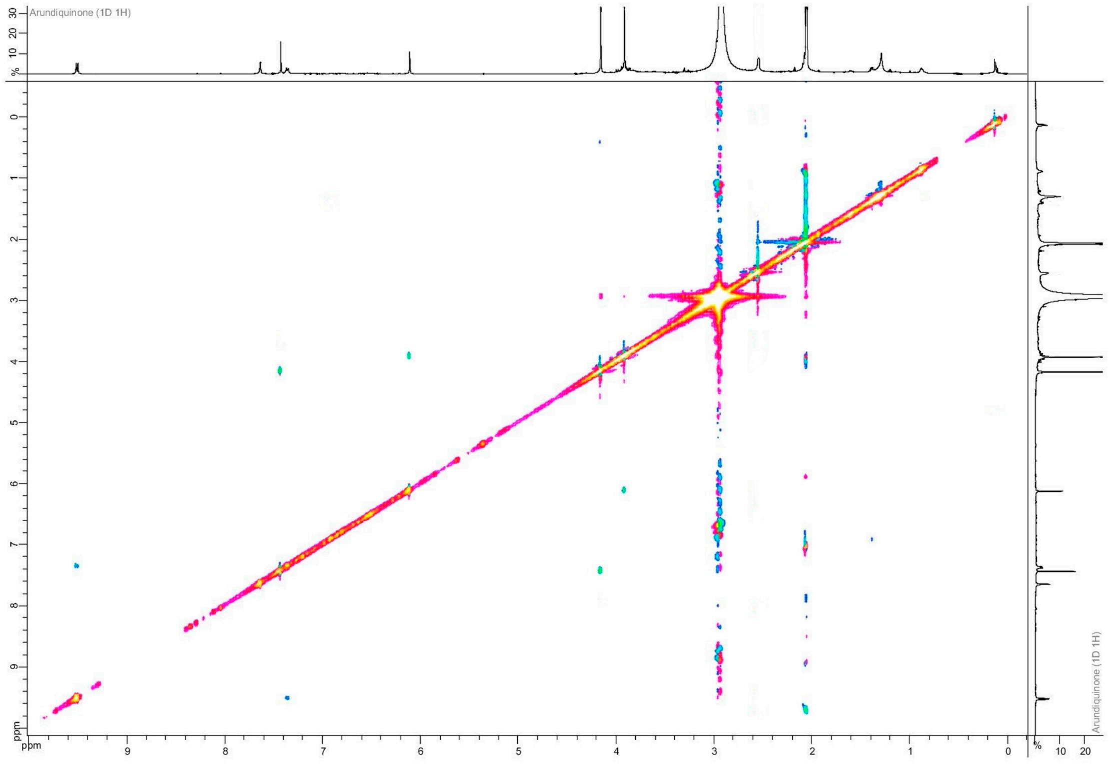

Figure S5. NOESY spectrum of arundiquinone (**1**).

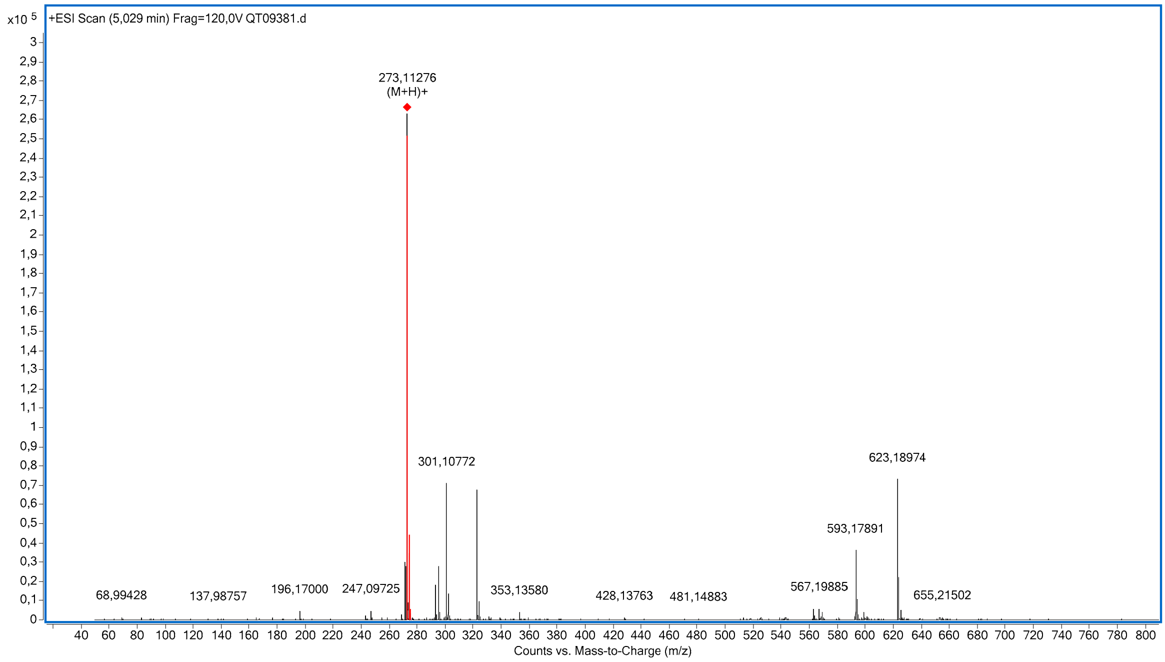

Figure S6. HRESIMS spectrum of arundiquinone (**1**).

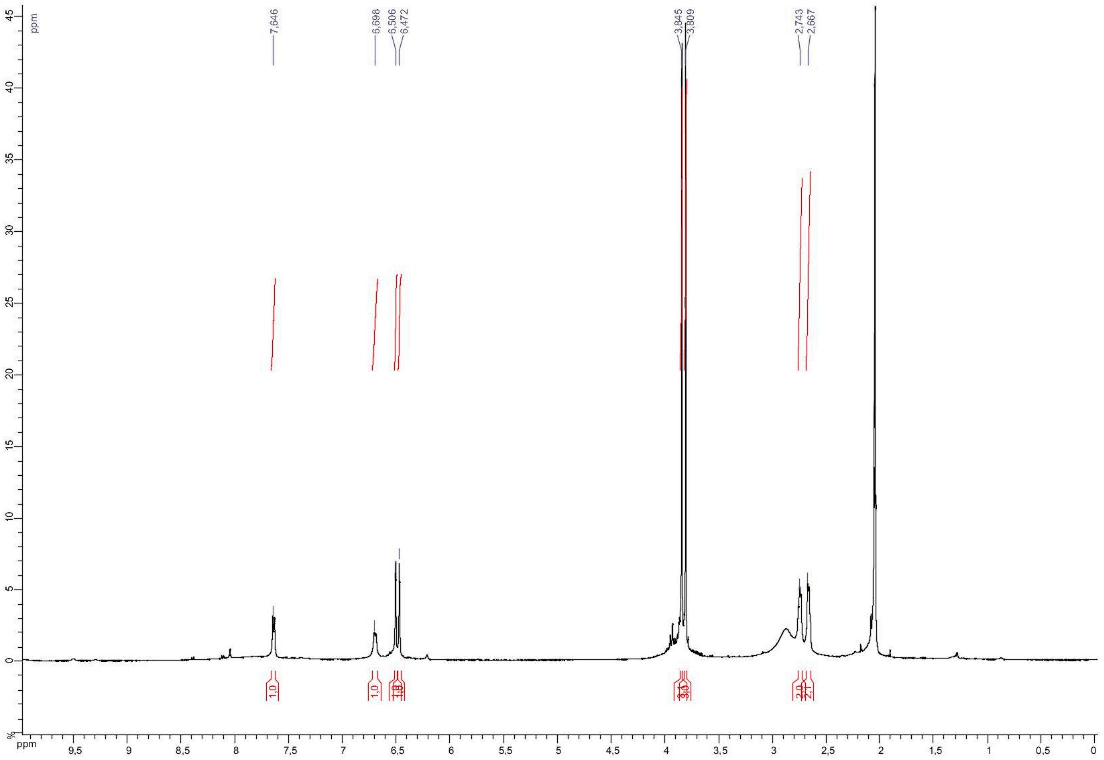

Figure S7. ^{1}H-NMR (500 MHz, acetone-d_6) spectrum of arundigramin (**2**).

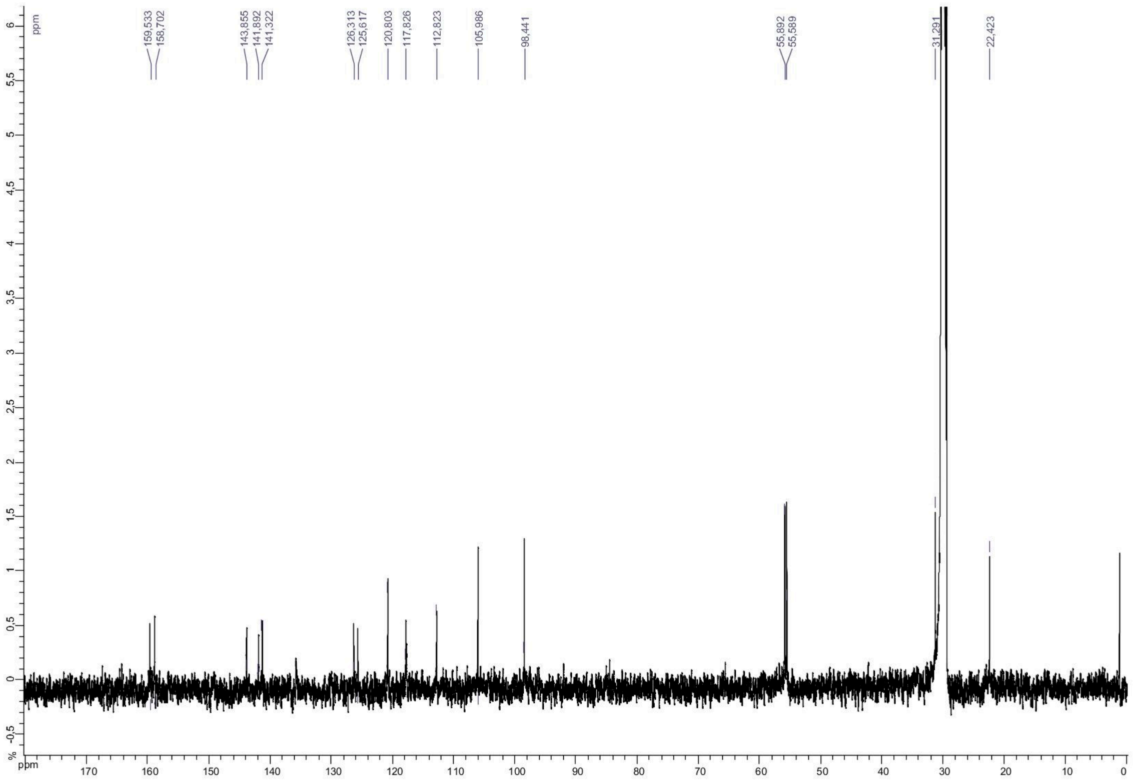

Figure S8. ^{13}C-NMR (125 MHz, acetone-d_6) spectrum of arundigramin (**2**).

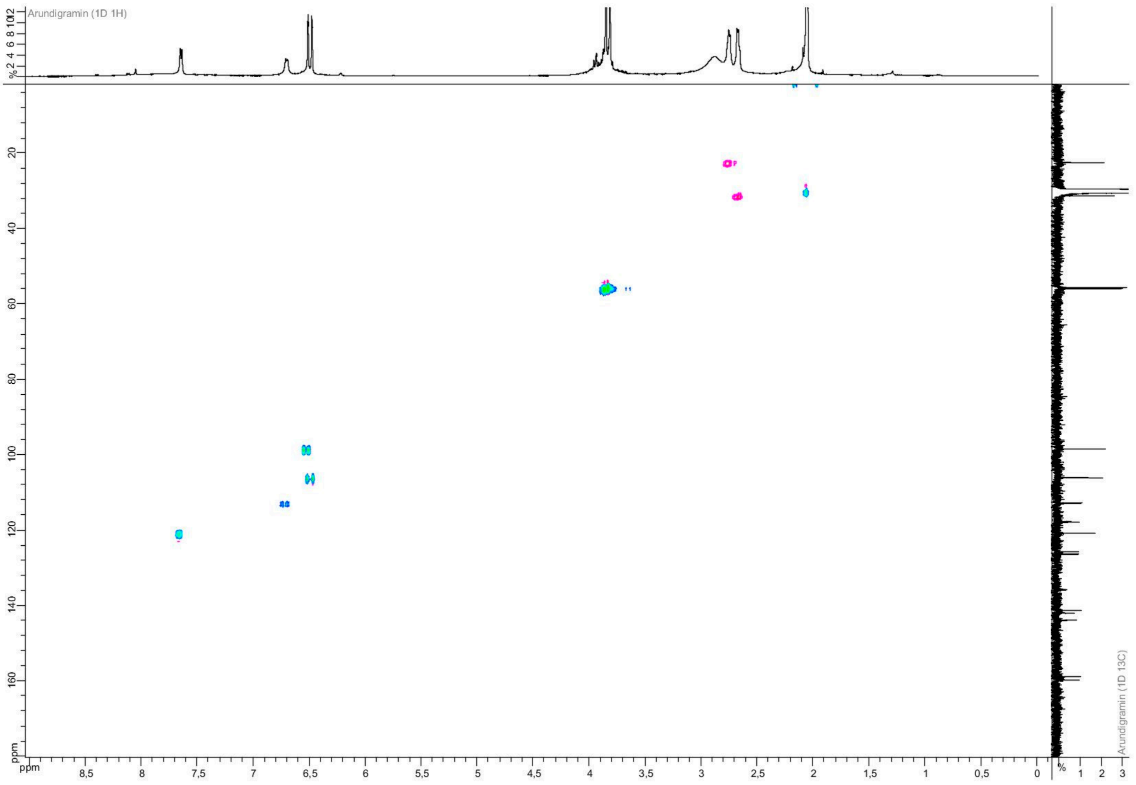

Figure S9. HSQC spectrum of arundigramin (**2**).

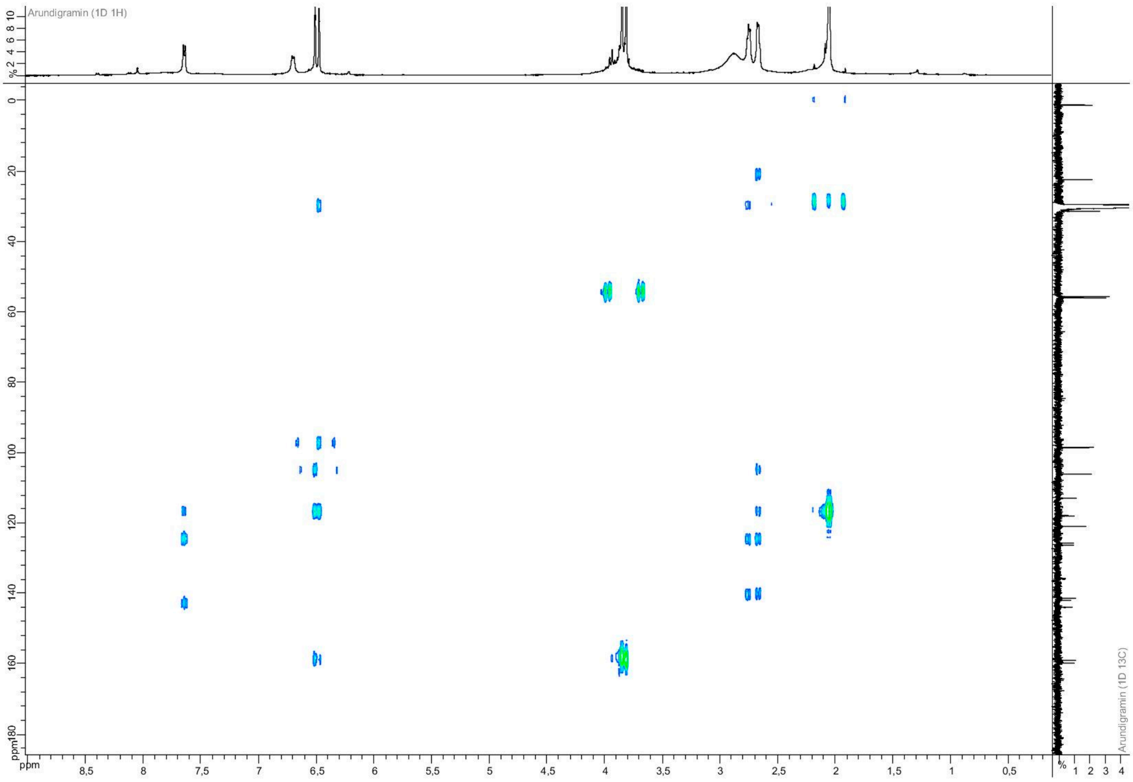

Figure S10. HMBC spectrum of arundigramin (**2**).

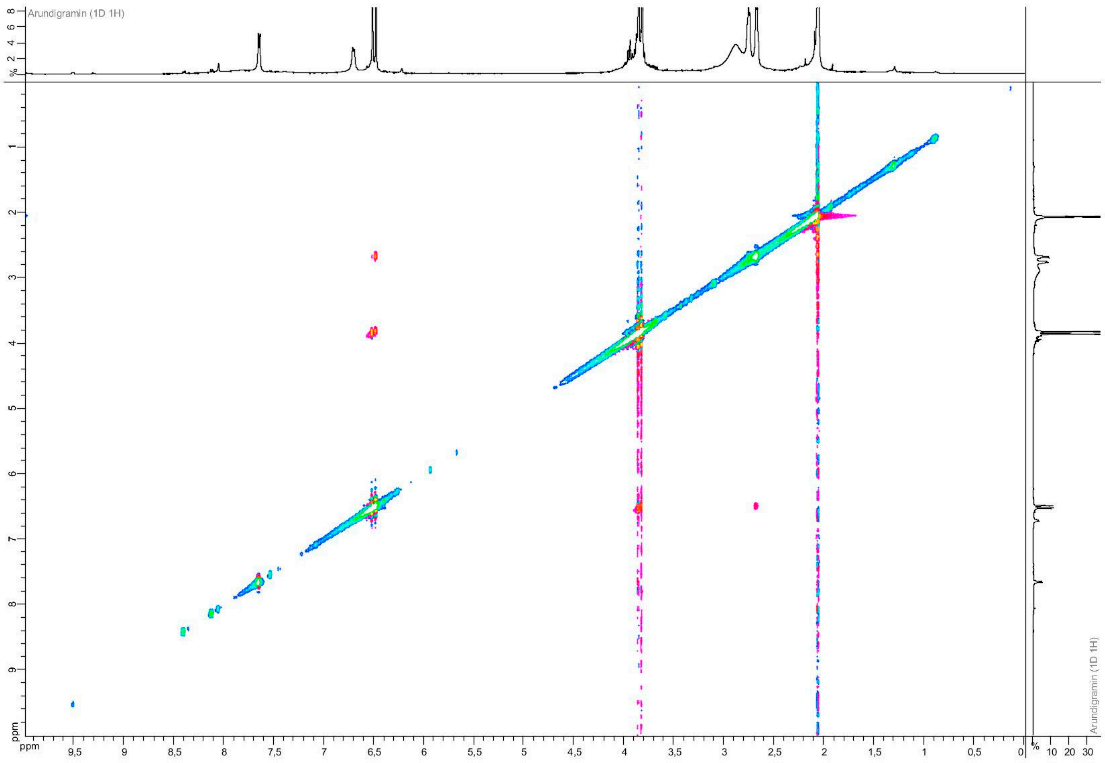

Figure S11. NOESY spectrum of arundigramin (**2**).

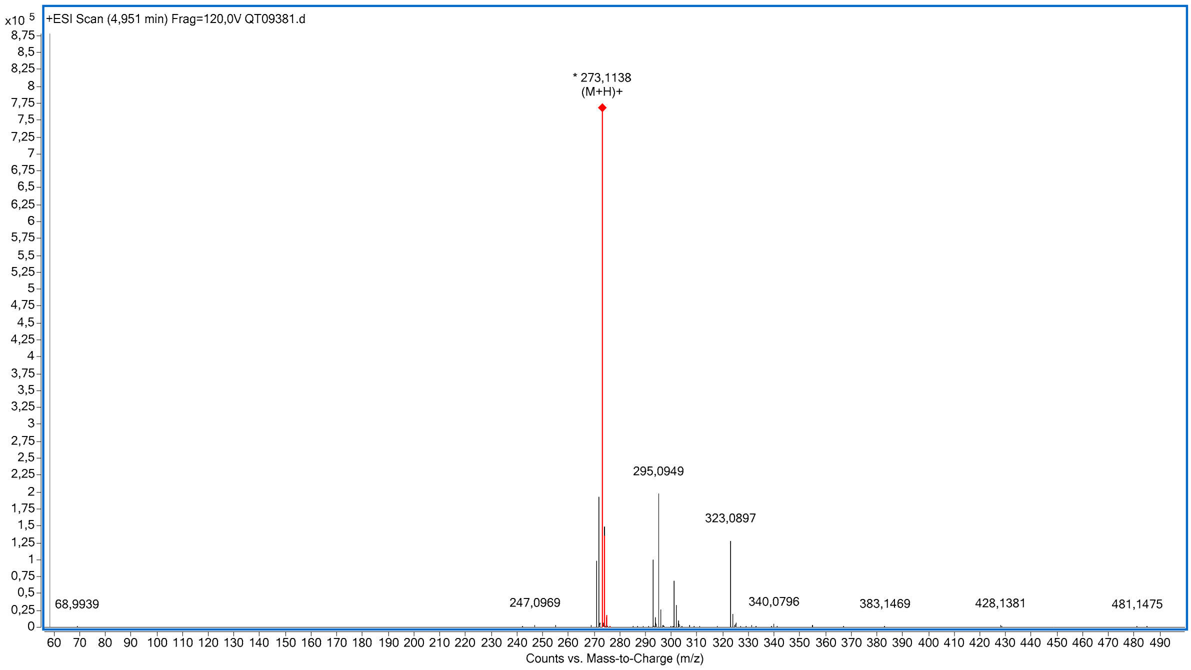

Figure S12. HRESIMS spectrum of arundigramin (**2**).
